# Analysis of Long Time Series of Summer Surface Urban Heat Island under the Missing-Filled Satellite Data Scenario

**DOI:** 10.3390/s23229206

**Published:** 2023-11-16

**Authors:** Jiamin Luo, Yuan Yao, Qiuyan Yin

**Affiliations:** 1 School of Architecture and Civil Engineering, Chengdu University, Chengdu 610106, China; luojiamin347@aliyun.com (J.L.); yqyyqy_171@aliyun.com (Q.Y.); 2 Key Laboratory of Pattern Recognition and Intelligent Information Processing of Sichuan Province, Chengdu University, Chengdu 610106, China; 3 State Key Laboratory of Resources and Environment Information System, Institute of Geographical Sciences and Natural Resources Research, Chinese Academy of Sciences, Beijing 100101, China

**Keywords:** surface urban heat island, land surface temperature, spatiotemporal fusion, spatial downscaling

## Abstract

Surface urban heat islands (SUHIs) are mostly an urban ecological issue. There is a growing demand for the quantification of the SUHI effect, and for its optimization to mitigate the increasing possible hazards caused by SUHI. Satellite-derived land surface temperature (LST) is an important indicator for quantifying SUHIs with frequent coverage. Current LST data with high spatiotemporal resolution is still lacking due to no single satellite sensor that can resolve the trade-off between spatial and temporal resolutions and this greatly limits its applications. To address this issue, we propose a multiscale geographically weighted regression (MGWR) coupling the comprehensive, flexible, spatiotemporal data fusion (CFSDAF) method to generate a high-spatiotemporal-resolution LST dataset. We then analyzed the SUHI intensity (SUHII) in Chengdu City, a typical cloudy and rainy city in China, from 2002 to 2022. Finally, we selected thirteen potential driving factors of SUHIs and analyzed the relation between these thirteen influential drivers and SUHIIs. Results show that: (1) an MGWR outperforms classic methods for downscaling LST, namely geographically weighted regression (GWR) and thermal image sharpening (TsHARP); (2) compared to classic spatiotemporal fusion methods, our method produces more accurate predicted LST images (*R*^2^, RMSE, AAD values were in the range of 0.8103 to 0.9476, 1.0601 to 1.4974, 0.8455 to 1.3380); (3) the average summer daytime SUHII increased form 2.08 °C (suburban area as 50% of the urban area) and 2.32 °C (suburban area as 100% of the urban area) in 2002 to 4.93 °C and 5.07 °C, respectively, in 2022 over Chengdu City; and (4) the anthropogenic activity drivers have a higher relative influence on SUHII than other drivers. Therefore, anthropogenic activity driving factors should be considered with CO_2_ emissions and land use changes for urban planning to mitigate the SUHI effect.

## 1. Introduction

The surface urban heat island (SUHI)—a phenomenon in which the land surface temperature (LST) tends to be higher in urban center zones than surrounding suburban surfaces, is usually measured using satellite thermal remote sensing data [1,2]. The SUHI effect is one of the greatest concerns for its adverse impacts on air and water quality, energy consumption, and urban dwellers’ health during heat wave events [3]. The SUHI phenomenon has been observed worldwide, especially in developing countries such as China [4]. In the summer of 2022, China faced its most severe heatwave in over six decades [5]. The province of Sichuan, situated in western China experienced record-breaking temperatures. This exacerbated the challenges faced by urban residents, including power outages, which were compounded by a widespread drought that severely affected both food and factory production across the province [6,7]. Consequently, accurately quantifying the intensity of the SUHI effect (SUHII) and better understanding the driving factors has become imperative. These measures not only aid in assessing potential heat-related risks but also contribute to future city management strategies, guiding governmental decision-making [8].

LSTs retrieved from satellite thermal infrared (TIR) bands are key indicators for quantifying SUHIs [9]. Satellite remote sensing has supplied effective and unique methods for acquiring LST data with frequent coverage. However, adverse atmospheric conditions coupled with long revisit cycles have largely limited satellite-derived LST applications in urban thermal environments [10]. Especially in the most rainy and cloudy cities in China, the large missing rate of satellite data is a common and serious problem. For a single satellite sensor, a tradeoff occurs between spatial and temporal resolution. Thus, there is a significant requirement to develop a method capable of integrating remotely sensed data from diverse sensors to produce fine spatiotemporal resolution LSTs for a better understanding of SUHI dynamics [11]. In the last ten years, numerous methods for spatiotemporal fusion have been suggested to achieve high-resolution LSTs by combining the high spatial resolution and the high temporal frequency of diverse remote sensing data sources [12]. The available spatiotemporal data fusion approaches that have been experimentally tested on LST products are mainly classified into four categories: weighted function-based [13], unmixing-based [14], learning-based [15], and hybrid methods [16]. The spatiotemporal fusion technique for LST data offers the opportunity to further understand the SUHI phenomenon [17,18]. In the present review, the spatial and temporal adaptive reflectance fusion model (STARFM) [14], the enhanced STARFM (ESTARFM) method [19], the bilateral filter [9], the spatiotemporal adaptive data fusion algorithm (SADFAT) [20], and the spatiotemporal integrated temperature fusion model (STITFM) [21] in the weight function-based category; the pixel-based multi-spatial resolution adaptive fusion modeling framework (pMSRAFM) [22] in the unmixing-based category; the sparse-representation-based spatiotemporal reflectance fusion model (SPSFTM) [23] in the learning-based category; and the flexible spatiotemporal data fusion (FSDAF) method [24] in the hybrid category. Although great progress has been made, most of these spatiotemporal fusion methods are unable to accurately capture the spatial details of LSTs, predict abrupt events, and preserve the spatial continuity of LSTs within urban areas simultaneously [25]. The above methods have their own advantages and limitations. For example, the weight function-based category has the most methods developed, owing to high computation efficiency, simple parameters, and strong robustness. However, this category has weaknesses in the strong temporal variability in LSTs that makes them more sensitive to model parameters [16], particularly, the size of the moving window, and it is not feasible in heterogeneous urban areas. FSDAF can simultaneously predict dense time series LST data owing to its ability to predict abrupt changes and gradual change events, but also easily causes spatial discontinuity in urban LST data. Shi et al. [26] proposed a comprehensive flexible spatiotemporal data fusion (CFSDAF) method based on FSDAF and generated a high-spatiotemporal-resolution LST image, which can preserve the spatial continuity and spatial details of LST in urban areas. At present, the application of LST in urban environment studies requires more heat-related information at the urban district level with high spatial resolution [27]. However, high-resolution LSTs may also be derived from the Landsat series TIR channels (i.e., Landsat 5, 8, and 9) at about 100 m but remain far from meeting the needs for improving SUHI monitoring accuracy.

In this study, Chengdu, a typical cloudy and rainy city in southwestern China has been selected as the study area. Chengdu has very few satellite images that are available for use, due to the annual average number of 340 days that experience cloudy and rainy weather. The main purposes of this study were to (1) propose a multiscale geographically weighted regression (MGWR) coupling CFSDAF method to generate a 30 m spatial resolution and 8 days temporal resolution summer LST dataset from 2002 to 2022, and produce higher accuracy in urban areas compared to other traditional spatiotemporal fusion methods; and (2) perform quantitative analyses to investigate the influence of multiple nature-anthropogenic driving factors on the summer SUHII in Chengdu City.

## 2. Data and Methods

### 2.1. Study Area

**Figure 1:**
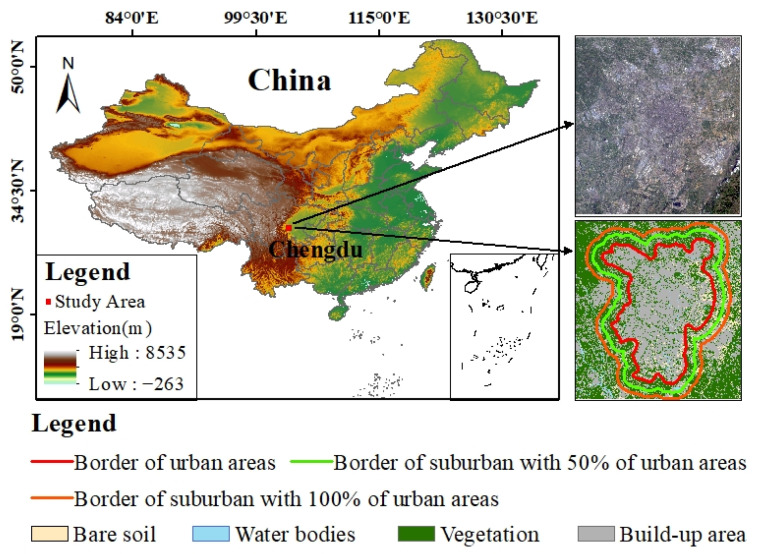
Location of the study area.

Chengdu, the capital city of Sichuan province and the sixth largest city in China (103°57′ E–104°20′ E, 31°15′ N–31°41′ N), has experienced rapid urbanization in the 21st century. In 2022, Chengdu’s gross domestic product (GDP) reached 2080 billion US dollars. The population of the city has exceeded 21.2 million people, 15.4 million of them living in urban areas. Rapid urbanization induces significant SUHI effects, especially in summer, which could lead to extreme heat events. From 5 to 24 August 2022, Chengdu experienced a record months-long heatwave, which exceeded 40 °C on seven days [28]. The long-term extreme heat phenomenon easily leads to air pollution and public health problems. This study focuses on an area of 60 km × 54 km in Chengdu, which covers the core urban area, a smaller suburban area with 50% of the urban area, and a larger suburban area with 100% of the urban area (Figure 1). One challenging problem for monitoring the summer SUHI effect in Chengdu is the large rate of missing satellite LST data owing to many cloudy and rainy days throughout the whole year.

### 2.2. Data Description and Preprocessing

**Figure 2:**
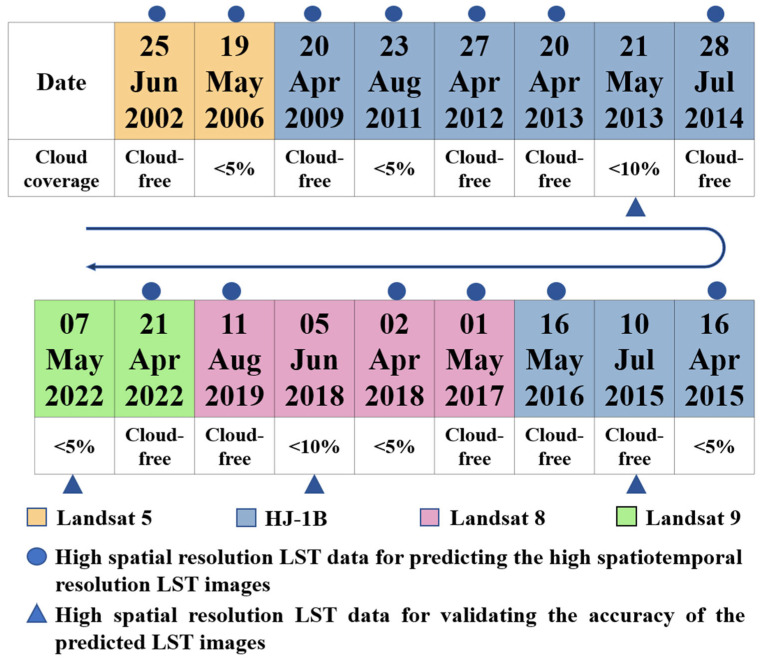
The selected high-spatial-resolution LST data for generating and evaluating the predicted LST results.

The proposed MGWR-CFSDAF method mainly needs, at least, a pair of high and low-spatial-resolution LST data on the prior date and one set of low-spatial-resolution LST data on the predicted date. In this study, due to limitations of cloudy and rainy weather, we can only select seventeen Landsat LST and HJ-1B LST data from 2002 to 2022 as the high spatiotemporal LST data through blending with low-spatial-resolution MODIS LST data for predicting high-spatiotemporal-resolution LST data. As shown in Figure 2, there are ten high-spatial-resolution LST images without clouds and seven LST images have a cloud cover ranging from 0% to 10%.

(1) Landsat 5/8/9 LST data. The Landsat thermal infrared (TIR) channels have a minimum 16-day revisit cycle and spatial resolution of about 100 m, as Landsat 5 collects TIR channel data at 120 m spatial resolution while Landsat 8/9 has two TIR bands at 100 m spatial resolution. Landsat images are available from the U.S. Geological Survey (http://earthexplorer.usgs.gov/, accessed on 20 January 2023). Radiometric calibration and atmospheric correction were performed. We retrieved Landsat LST data from Landsat 5 TIR band 6 and Landsat 8/9 TIRS band 10 using a generalized single-channel method. For details of the generalized single-channel method, please refer to Jimenez-Munoz and Sobrino [29]. The details of the Landsat, HJ-1B, and MODIS used in this study are summarized in Table 1.

(2) HJ-1B LST data. The HJ-1B images used in this study are level-2 output products and were obtained from the China Center for Resources Satellite Data and Application (https://data.cresda.cn/#/mapSearch/, accessed on 25 January 2023). The spatial resolution of the HJ-1B TIR band is 300 m with a 4-day revisit cycle [30]. The HJ-1B data were geometrically corrected using calibrated Landsat 8 images within the study area. The error was controlled within 0.5 pixels to meet the geometry correction requirements. Then, the HJ-1B data were radiometrically calibrated using calibration coefficients [31] to convert the digital number (DN) values of the raw HJ-1B images into satellite radiance images [32]. Finally, the ENVI-FLAASH module was used for atmospheric correction on each HJ-1B CCD image after radiometric calibration. In this research, LST data were retrieved from the thermal band IRS4 of the HJ-1B imagery using the single-channel algorithm. For a more comprehensive description of the single-channel algorithm, please refer to the work of Duan et al. [33].

(3) MODIS LST data. One kilometer spatial resolution Daily Terra MODIS daytime LST (MOD11A1) data and 8-day Terra MODIS daytime LST (MOD11A2) data were obtained using the generalized split-window algorithm from the Geospatial Data Cloud (http://www.gscloud.cn/, accessed on 28 January 2023). Numerous research findings indicate that the root mean square error (RMSE) of the MODIS LST data are within 2.0 K and exhibit high accuracy in major global cities [34]. MODIS LST data were re-projected to the same coordinate system as Landsat and HJ-1B using MODIS Reprojection Tools (MRT). Finally, we utilized the quality control band within MOD11A1 and MOD11A2 to identify pixels affected by cloud contamination, with the purpose of excluding them from subsequent analysis.

(4) In situ LST. In situ hourly LST data were collected from Chengdu Meteorological Office’s 7 weather stations distributed across Chengdu City in summer (April to September) from 2002 to 2022. In situ LSTs were collected based on SI-111 infrared radiometers with an accuracy of ±0.2 K.

(5) Potential driving factors of SUHII. To explore the potential driving factors of SUHII in Chengdu City, thirteen driving factors were selected in this study and divided into four types: the satellite precipitation product (PRE), wind speed (WS), relative humidity (RH), and white sky albedo (WSA) form the climate types; nighttime light index (NLI), perpendicular impervious surface index (PISI), and PM_2.5_ form the anthropogenic activity types; population counts (POP), population density (PD), and gross domestic products (GDP) form the population shift types; and enhanced vegetation index (EVI), bare-soil index (BI), and normalized difference water index (NDWI) form the natural land surfaces types. Table 2 shows the potential driving factors based on available data selected in this study.

### 2.3. Generating High-Spatiotemporal-Resolution LST for SUHI Monitoring

**Figure 3:**
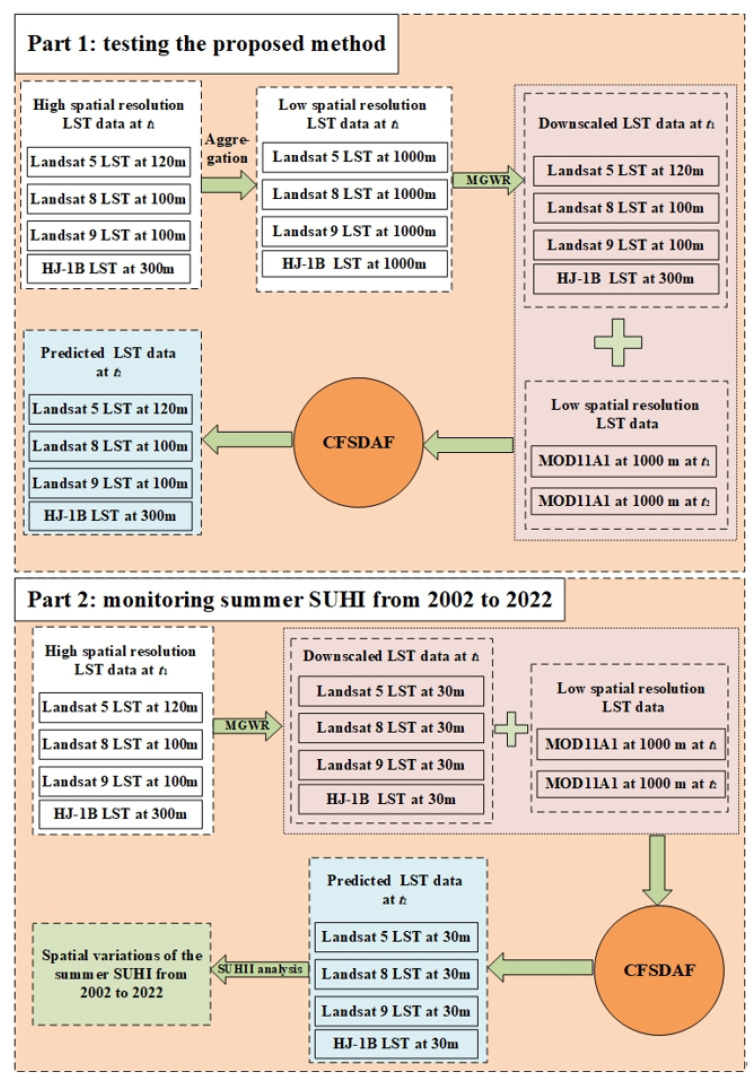
Flowchart of testing the proposed method and monitoring daytime SUHI.

In this study, in order to generate the 30 m spatial resolution and 8-day temporal resolution summer LST dataset from 2002 to 2022, a multiscale geographically weighted regression MGWR coupling CFSDAF method was proposed. The implementation consists of testing the proposed method part and monitoring the summer daytime SUHI part (Figure 3). If the performance of the first part is better, we can conduct the next part.

In the first part (testing the proposed method), both downscaled high-spatial-resolution LST data using the MGWR model and MOD11A1 data captured on 20 April 2013, 16 April 2015, 2 April 2018, and 21 April 2022 were used as the base time (*t*_1_) for the proposed method. While the other MOD11A1 data captured on 21 May 2013, 10 July 2015, 5 June 2018, and 7 May 2022 were used as the prediction time (*t*2) input base data for predicting the high-spatial-resolution LST at *t*2. In the following part, we call LST directly from MODIS, HJ-1B, Landsat as “observed LST”, and LST derived from the proposed method of other spatiotemporal fusion methods as “predicted LST”. The coefficient of determination (*R*^2^), the RMSE and the absolute average difference (AAD), were computed between the predicted LST images and the observed LST images to validate the accuracy of the predicted LST data.

In the second part (monitoring summer daytime SUHI from 2002 to 2022), we selected thirteen pairs of downscaled LST with 30 m spatial resolution and MOD11A1 data with 1000 m spatial resolution during the same period as the input base LST data at *t*1 (Table 1). Afterwards, MOD11A2 at *t*2 was used to fuse the predicted LST datasets with a temporal resolution of 8 days and 30 m spatial resolution. Finally, the predicted summer daytime LSTs were averaged and used to monitor the summer SUHI from 2002 to 2022 in Chengdu City.

#### 2.3.1. Downscaling LST Using MGWR

Classical geographically weighted regression (GWR) as a downscaling method cannot capture the spatial non-stationary relationship between LSTs and environmental variables [70]. Unlike GWR, MGWR can build a nonstationary relationship between LSTs and multiple environmental variables [71]. MGWR is introduced to analyze the scale differences in normalized vegetation index (NDVI), digital elevation model (DEM), slope, and aspect on the spatial pattern of LSTs. The MGWR model was employed to downscale LST changes from low-resolution LST data to high-resolution LST data. The mathematical expression of the MGWR is as follows [72]:

(1) $$Y_{i}=\beta_{\mathrm{bw}0}\left(\mu_{i},v_{i}\right)+\sum_{j=1}^{n}\beta_{\mathrm{bwj}}\left(\mu_{i},v_{i}\right)X_{ij}+\varepsilon_{i}$$

where $Y_{i}$ is the predicted values of dependent variable (LST in our case) and *i* = 1, 2,3, …, *n*; and $\beta_{bw0}$ is a the intercept at optimal bandwidth. $X_{ij}$ represents the jth predictor variable with spatially varying regression coefficient ($\beta_{bwj}$) over spatial locations ($\mu_{i},v_{i}$). The error term in the model is represented by $\varepsilon_{i}$.

**Figure 4:**
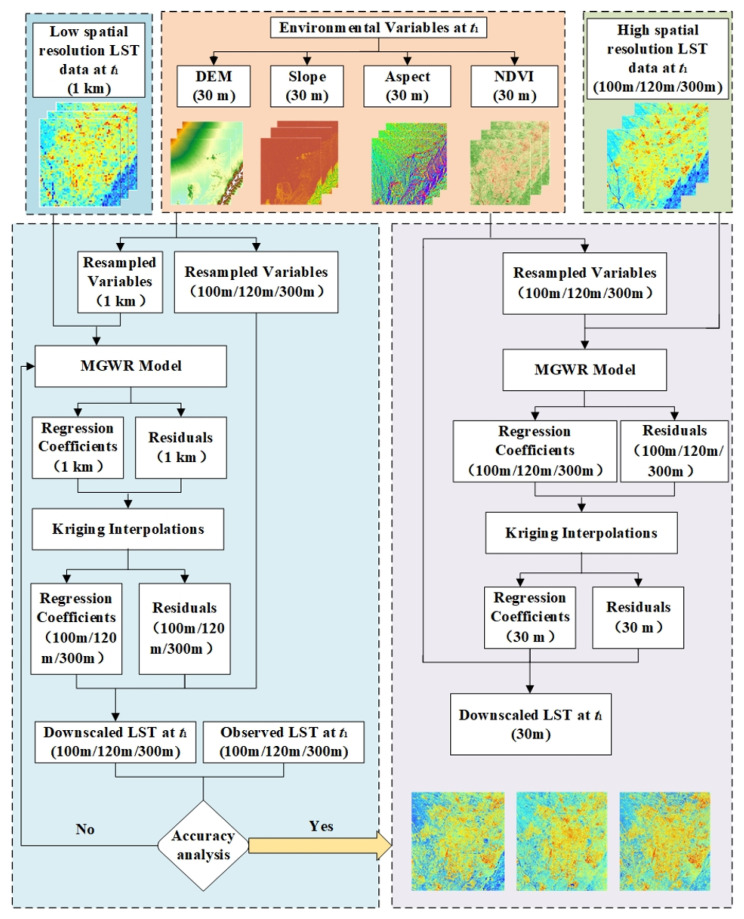
Flowchart of testing LST downscaling procedure based on MGWR.

The specific steps of MGWR-based LST downscaling method are shown in Figure 4 and are summarized as follows:

(1) LST retrieval from Landsat 5, Landsat 8, Landsat 9 and HJ-1B were aggregated to the spatial resolution of 1000 m. NDVI, DEM, slope, and aspect were extracted at a 30 m spatial resolution based on the Landsat imagery, HJ-1B imagery, and other auxiliary data, whereas these environmental variables were aggregated to the spatial resolution 100 m, 120 m, 300 m, and 1000 m, respectively.

(2) MGWR was used to establish a nonstationary relationship between *LST*_1000_, and *NDVI*_1000_ as well as *DEM*_1000_, *Slope*_1000_, and *Aspect*_1000_, which can be expressed as

(2) $${\mathrm{LST}}_{1000}=f\left({\mathrm{NDVI}}_{1000},{\mathrm{DEM}}_{1000},{\mathrm{Slope}}_{1000},{\mathrm{Aspect}}_{1000}\right)$$

where ${\mathrm{LST}}_{1000}$ is the LST estimated by the scale conversion function at 1000 m spatial resolution scale; NDVI_1000_, DEM_1000_, Slope_1000_, and Aspect_1000_ are environmental variables at 1000 m spatial resolution; $f\left(.\right)$ is the MGWR converts the auxiliary variables to simulate LST.

(3) Influenced by soil moisture and other physical parameters, it is difficult to fully reflect the spatial heterogeneity of LST, which is manifested as LST residual information at low-spatial-resolution scales:

(3) $$\Delta LST_{s}={\mathrm{LST}}_{s}-\bar{{\mathrm{LST}}_{s}}$$

where $\Delta LST_{s}$ is the LST transformation residual at 1000 m spatial resolution; $\bar{{\mathrm{LST}}_{s}}$ is the LST data estimated by the MGWR; and ${\mathrm{LST}}_{s}$ is the LST at a 1000 m spatial resolution. Assuming that the residuals are uniformly spatially distributed, we further interpolated the transformed residuals to a resolution of 120 m (Landsat 5 LST), 100 m (Landsat 8/9 LST), and 300 m (HJ-1B LST) using ordinary kriging interpolation [51].

(4) $f\left(.\right)$ established at low-spatial-resolution scales is still applicable to other spatial resolutions according to the constant relational scale’ principle. Combined with the transformed residuals after spatial interpolation, the LST data downscaled to a 100 m, 120 m, and 300 m spatial resolution, which is

(4) $${\mathrm{LST}}_{100}=f\left({\mathrm{NDVI}}_{100},{\mathrm{DEM}}_{100},{\mathrm{Slope}}_{100},{\mathrm{Aspect}}_{100}\right)+\Delta LST_{s1}$$

(5) $${\mathrm{LST}}_{120}=f\left({\mathrm{NDVI}}_{120},{\mathrm{DEM}}_{120},{\mathrm{Slope}}_{120},{\mathrm{Aspect}}_{120}\right)+\Delta LST_{s2}$$

(6) $${\mathrm{LST}}_{300}=f\left({\mathrm{NDVI}}_{300},{\mathrm{DEM}}_{300},{\mathrm{Slope}}_{300},{\mathrm{Aspect}}_{300}\right)+\Delta LST_{s3}$$

where ${\mathrm{LST}}_{100}$, ${\mathrm{LST}}_{120}$, and ${\mathrm{LST}}_{300}$ are the downscaled LST data at a spatial resolution of 100 m, 120 m, and 300 m, respectively. ${\mathrm{NDVI}}_{100}$, ${\mathrm{NDVI}}_{120}$, ${\mathrm{NDVI}}_{300}$, ${\mathrm{DEM}}_{100}$, ${\mathrm{DEM}}_{120}$, ${\mathrm{DEM}}_{300}$, ${\mathrm{Slope}}_{100}$, ${\mathrm{Slope}}_{120}$, ${\mathrm{Slope}}_{300}$, ${\mathrm{Aspect}}_{100}$, ${\mathrm{Aspect}}_{120}$, and ${\mathrm{Aspect}}_{300}$ are the 100 m, 120 m, and 300 m, respectively, environmental variables after spatial aggregation; and $\Delta LST_{s1}$, $\Delta LST_{s2}$, and $\Delta LST_{s3}$ are the 100 m, 120 m, and 300 m spatial resolution conversion residual after spatial interpolation.

(5) If the validation of the MGWR possess is good, then we will perform upscaled LST at 1000 m spatial resolution to 100 m, 120 m, and 300 m. MGWR was used to downscale observed LST from 100 m, 120 m, and 300 m to 30 m.

#### 2.3.2. Implementation of the Proposed Method

Figure 3 presents a detailed producer of the proposed method. In this study, the CFSDAF method was used to fuse high-spatiotemporal-resolution LST images in the study area, in order to monitor summer SUHII, by combining the MOD11A1, MOD11A2, and downscaled MGWR LST images. The calculation process can be expressed as follows:

(7) $${\tilde{\mathrm{LST}}}_{t_{2}}\left(x_{ij},y_{ij}\right)={\mathrm{LST}}_{t_{1}}\left(x_{ij},y_{ij}\right)+\sum_{k=1}^{n}\left[w_{k}\times \Delta LST\left(x_{ij},y_{ij}\right)\right]$$

where ${\tilde{\mathrm{LST}}}_{t_{2}}\left(x_{ij},y_{ij}\right)$ represents the predicted high-resolution LST image at prediction data $t_{2}$; ${\mathrm{LST}}_{t_{1}}\left(x_{ij},y_{ij}\right)$ represents the high-resolution LST data at base time $t_{1}$; *k* is the *k*th similar pixel; *n* is the number of similar pixels for central pixel in a single window; and $\Delta LST\left(x_{ij},y_{ij}\right)$ is the prediction of the total change of the target pixel $\left(x_{ij},y_{ij}\right)$ between $t_{1}$ and $t_{2}$.

In this study, CFSDAF mainly includes the following six steps: (1) adjust the differences between high-spatial-resolution LST and low-spatial-resolution LST and high-spatial-resolution LST; (2) classify high-spatial-resolution LST after extracting the endmembers; (3) obtain the temporal increments by the linear equation of spatial unmixing process; (4) obtain the spatial increments by inverse distance weighting (IDW) interpolation; (5) integrate the spatial and temporal increments; and (6) obtain the LST prediction by the information of neighborhood. For more detailed steps of the CFSDAF model kindly refer to previous studies [26].

#### 2.3.3. SUHII Analysis

In this study, SUHII is the LST difference between urban (${\mathrm{LST}}_{\mathrm{urban}}$) and suburban areas (${\mathrm{LST}}_{\mathrm{suburban}}$) using the fused high spatiotemporal resolution summer LST dataset from 2002 to 2022 over Chengdu City. The formula is as follows [8]:

(8) $${\mathrm{SUHII}=\mathrm{LST}}_{urban}-{\mathrm{LST}}_{suburban}$$

#### 2.3.4. Boosted Regression Tree Model

The application of the machine learning statistical model, boosted regression tree (BRT), is employed to investigate the influences of thirteen potential driving factors on SUHI. The BRT model exhibits strong learning capabilities and adaptability to diverse data formats, even when handling complex data, without necessitating the consideration of interactions or correlations among independent variables. Furthermore, it offers significant advantages in exploring interactions between complex factors and making forecasts. The BRT model has found successful applications in a wide range of fields, including urban expansion, ecological modeling, and environmental science [73,74,75].

In this paper, the gbm package with the statistical programming software R (version 3.3.2) was used to analyze the contribution of potential driving factors to SUHI. The dependent variables are the SUHII, and the independent variables are the thirteen driving factors. The BRT model is a supervised learning method; three parameters were specified after testing. In this study, the learning rate, bagging fraction, and decision tree complexity were 0.01, 0.5 and 5, respectively. In this study, this model extracted 50% of the data points for training, with 50% of the data used to fit thirteen driving factors and the first regression tree is SUHI.

## 3. Results

### 3.1. Land Cover Classification

In order to monitor the SUHII in Chengdu, defining the urban and suburban areas was the first step. In this study, support vector machine (SVM), as one of the machine learning algorithms, was used for image classification [76]. Cloud-free Landsat 5 images were acquired on 25 June 2002 and 9 May 2006, HJ-1B images were acquired on 20 April 2009, 23 August 2011, 27 April 2012, 20 April 2013, 28 July 2014, 16 April 2015, and 16 May 2016, Landsat 8 images were acquired on 1 May 2017, 2 April 2018, and 11 August 2019, and a Landsat 9 image was acquired on 21 April 2022. The landcover maps from 2002 to 2009 were classified using SVM based on the above cloud-free data. Land cover types are mainly built-up areas, water bodies, vegetation, and bare soil, which are typical in urban areas. Using Google Earth, we randomly chose 1600 sample points, 400 for each type, for the accuracy assessment (Figure 1). We also compared the performance of three machine learning classifiers—SVM, artificial neural networks (ANN), and maximum likelihood classification (MLC). Table 3 illustrates the overall accuracy and kappa coefficients. The result showed that SVM performed better than ANN and MLC.

**Figure 5:**
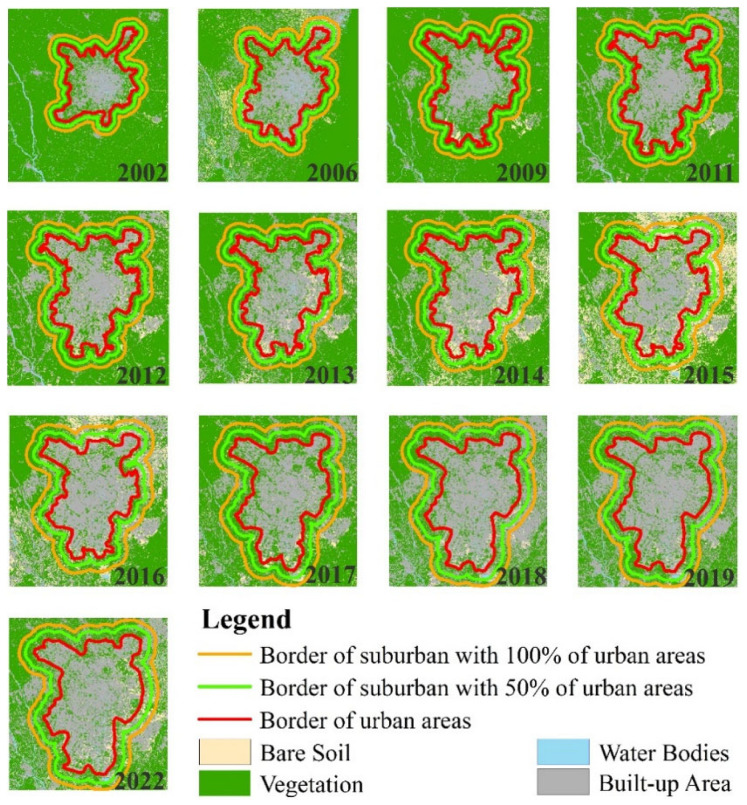
The delineation of urban and suburban areas over Chengdu City from 2002 to 2022.

Due to Chengdu being the sixth-largest city in China, its administrative boundaries encompass not only urban areas but also extensive suburban regions, which do not align with the requirements of the SUHI study. Therefore, in this study, urban and suburban areas were separated according to four land cover classification types from landcover maps. An urban area is defined as a high-intensity and densely occupied areas near a built-up area. After the urban area is determined, a suburban area is defined as the buffer zone that includes a smaller suburban area (50% of the urban area) and a larger suburban area (100% of the urban area) around the urban area (Figure 5).

### 3.2. Testing the Proposed Method

**Figure 6:**
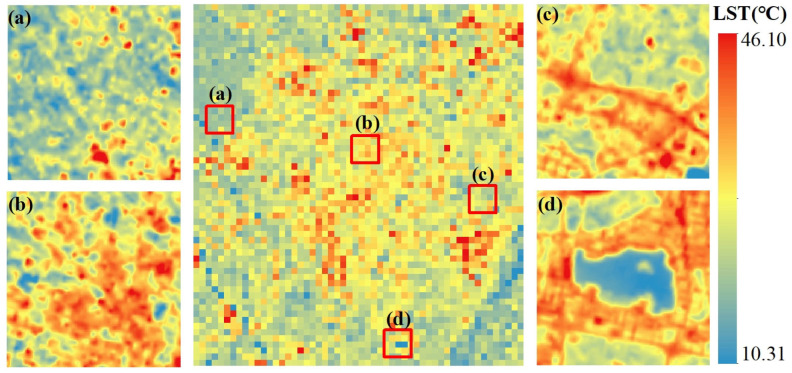
The 1000 m aggregated Landsat 9 LST on 21 April 2022 of (**a**) 100 m downscaled LST of vegetation, (**b**) 100 m downscaled LST of built-up area, (**c**) 100 m downscaled LST of brae soil, and (**d**) 100 m downscaled LST of water body.

MGWR is an extension of the generalized linear regression, with NDVI, DEM, slope, and aspect as the nominated environmental variable set that is highly LST-related. Firstly, high-spatial-resolution LST at the *t*1, such as Landsat 9 LST observed on 21 April 2022 (Figure 6) with 100 m resolution, was aggregated to 1000 m. The LST downscaling results from MGWR from 1000 m to 100 m is shown in Figure 6, where four kinds of subareas, notably subarea (Figure 6a) in vegetation, subarea (Figure 6b) in built-up area, subarea (Figure 6c) in bare soil area, and subarea (Figure 6d) in waterbody area, were used to show the LST downscaling performance using MGWR. Visually, MGWR can extract more spatial texture information from land surface temperature data, effectively revealing temperature distribution variations within similar land cover types.

To assess the accuracy of the method of MGWR in downscaling LST, GWR and thermal image sharpening (TsHARP), which possess the advantage of LST downscaling, were also used in this study. RMSE and the mean error (ME) as the evaluation metrics were used to quantitatively evaluate the performance of the three downscaling methods, which is shown in Table 4. We can see that the MGWR possesses lower RMSE and ME compared to GWR and TsHARP; it shows MGWR produces higher LST downscaling accuracy than other methods from 2002 to 2022 in the study area.

**Figure 7:**
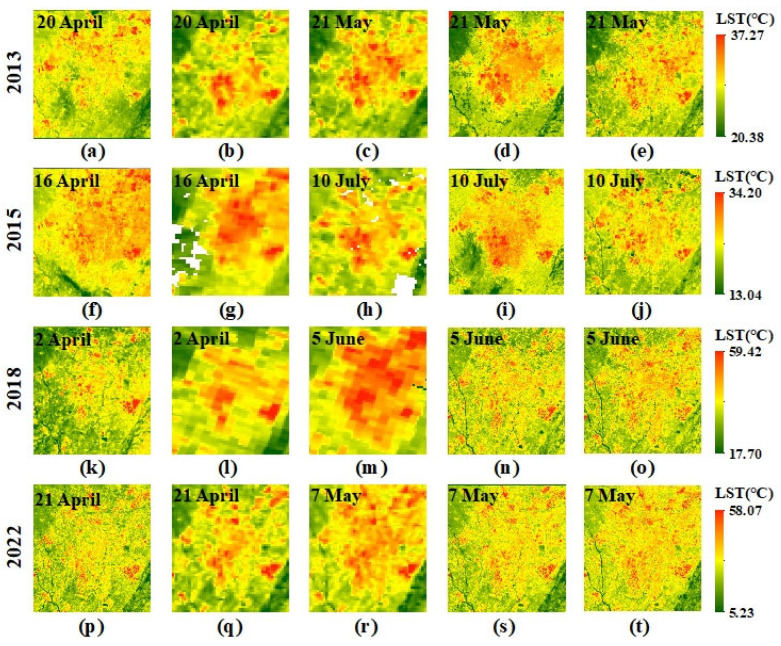
Spatial distributions of LST: (**a**) 300 m downscaled LST at *t*_1_ on 20 April 2013; (**b**) 1000 m MOD11A1 at *t*_1_ on 20 April 2013; (**c**) 1000 m MOD11A1 at *t*_2_ on 21 May 2013; (**d**) 300 m predicted LST on 21 May 2013; (**e**) observed LST at *t*_2_ on 21 May 2013; (**f**) 300 m downscaled LST at *t*_1_ on 16 April 2015; (**g**) 1000 m MOD11A1 at *t*_1_ on 16 April 2015; (**h**) 1000 m MOD11A1 at *t*_2_ on 10 July 2015; (**i**) 300 m predicted LST on 10 July 2015; (**j**) observed LST at *t*_2_ on 10 July 2015; (**k**) 100 m downscaled LST at *t*_1_ on 2 April 2018; (**l**) 1000 m MOD11A1 at *t*_1_ on 2 April 2018; (**m**) 1000 m MOD11A1 at *t*_2_ on 5 June 2018; (**n**) 100 m predicted LST on 5 June 2018; (**o**) observed LST at *t*_2_ on 5 June 2018; (**p**) 100 m downscaled LST at *t*_1_ on 21 April 2022; (**q**) 1000 m MOD11A1 at *t*_1_ on 21 April 2022; (**r**) 1000 m MOD11A1 at *t*_2_ on 7 May 2022; (**s**) 100 m predicted LST on 7 May 2022; (**t**) observed LST at *t*_2_ on 7 May 2022.

Therefore, the downscaled LST images and MOD11A1 at *t*_1_ could be used as the LST base data of CFSDAF for predicting the high-spatial-resolution LST data at *t*_2_. The next step is testing the performance of the proposed method in the first part (Figure 3). Figure 7a,f,k,p was downscaled using MGWR LST at *t*_1_ on 20 April 2013, 16 April 2015, 2 April 2018, and 21 April 2022, respectively. Figure 7b,g,i,q was MOD11A1 as the similar time at *t*_1_. Figure 7c,h,m,r was the MOD11A1 data at *t*_2_ on 21 May 2013, 10 July 2015, 5 June 2018, and 7 May 2022, respectively, for predicting the LST data at 100 m and 300 m spatial resolution on the same predicted date at *t*_2_ (Figure 7d,i,n,s). The observed LST data at *t*_2_ (Figure 7e,j,o,t) can be used to evaluate the predicted LST results as the similar time at *t*_2_.

**Figure 8:**
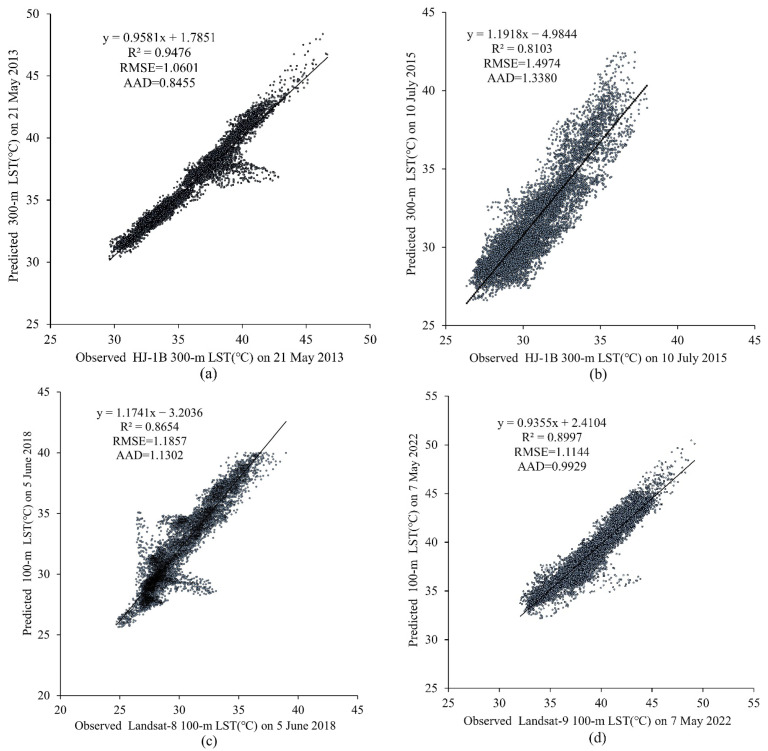
Scatter plots of the relation between observed LST and predicted LST image for: (**a**) 21 May 2013, (**b**)10 July 2015, (**c**) 5 June 2018, and (**d**) 7 May 2022.

Figure 8 shows scatter plots of correlations between observed LST and predicted LST on 21 May 2013, 10 July 2015, 5 June 2018, and 7 May 2022. We can see some of the scatters deviate a lot from the fitted line owing to the predicted LST images being affected by weather conditions like thin clouds and fog. However, the accuracy assessment shows that the *R*^2^ ranges from 0.8103 to 0.9476, RMSE from 1.0601 to 1.4974, and AAD from 0.8455 to 1.3380 on the same dates, which proves the proposed method has a better performance for predicting the high-spatiotemporal-resolution LST data.

**Figure 9:**
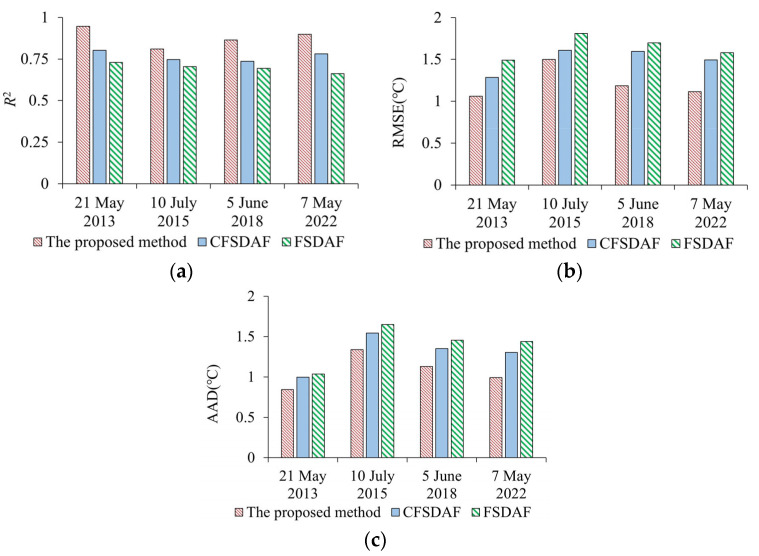
Comparison of the predicted LSTs using the proposed method and CFSDAF, FSDAF. (**a**) the coefficient of determination (*R*^2^); (**b**) the root mean square error (RMSE); (**c**) the absolute average difference (AAD).

In addition, CFSDAF and FSDAF were used to evaluate the performance of the predicted LST results using the proposed method (Figure 9). The *R*^2^, RMSE, and AAD between the predicted LST and the observed LST on 21 May 2013, 10 July 2015, 5 June 2018, and 7 May 2022 show that the proposed method can be used to improve the fusion accuracy of high-spatial-resolution LST. The proposed method, with higher accuracy than CFSDAF and FSDAF on different dates, which shows the performance of the spatiotemporal fusion model, is more sensitive to the spatial resolution scale.

### 3.3. Monitoring Summer SUHII from 2002 to 2022

**Figure 10:**
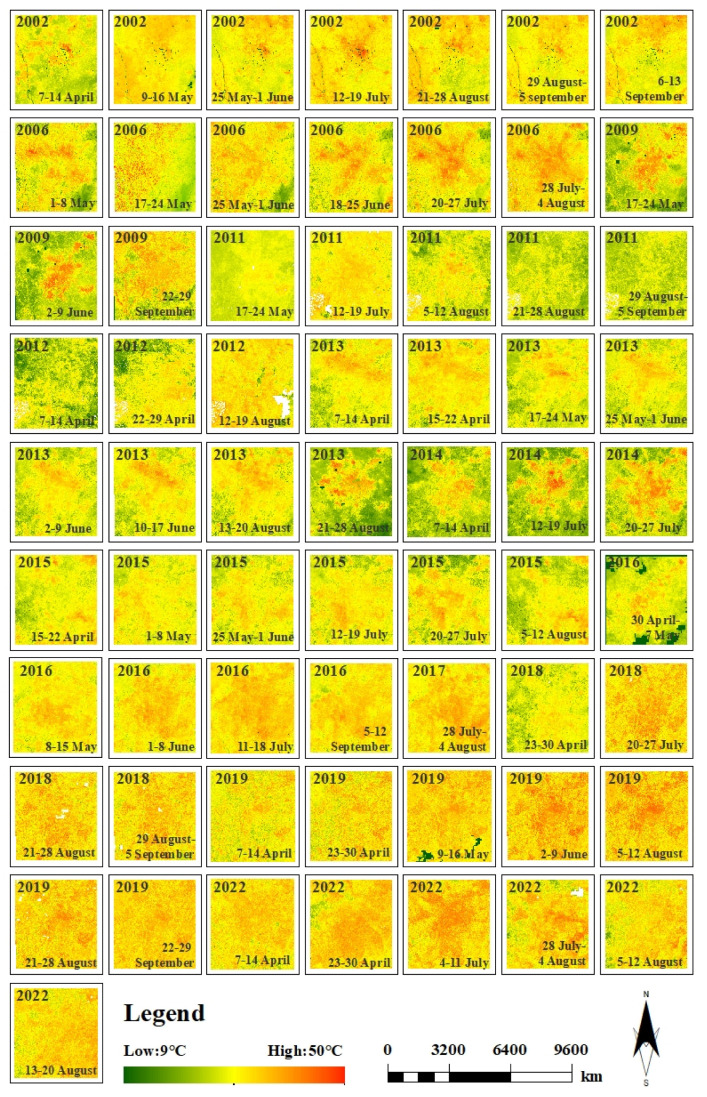
Spatial distributions of 30-m predicted LST using the proposed method.

In this study, the proposed method has a better performance for predicting the high-spatiotemporal-resolution LST data in the first part (testing the proposed method), which suggests that we can conduct the next step to predict high-spatiotemporal-resolution LST data using the proposed method with 30 m spatial resolution and 8-day temporal resolution in summer for monitoring summer SUHII in Chengdu City from 2002 to 2022 (Figure 10). Since there is no real satellite-derived LST data at 30 m spatial resolution, in situ LST data were used to validate the predicted LST results. In addition, the CFSDAF model, the FSDAF model, and the observed MOD11A2 at *t*_2_ were also used to evaluate the performance of the proposed method. Table 5 shows the proposed method can produce higher accuracy predictions of high-spatiotemporal-resolution LST data than other spatiotemporal fusion methods.

**Figure 11:**
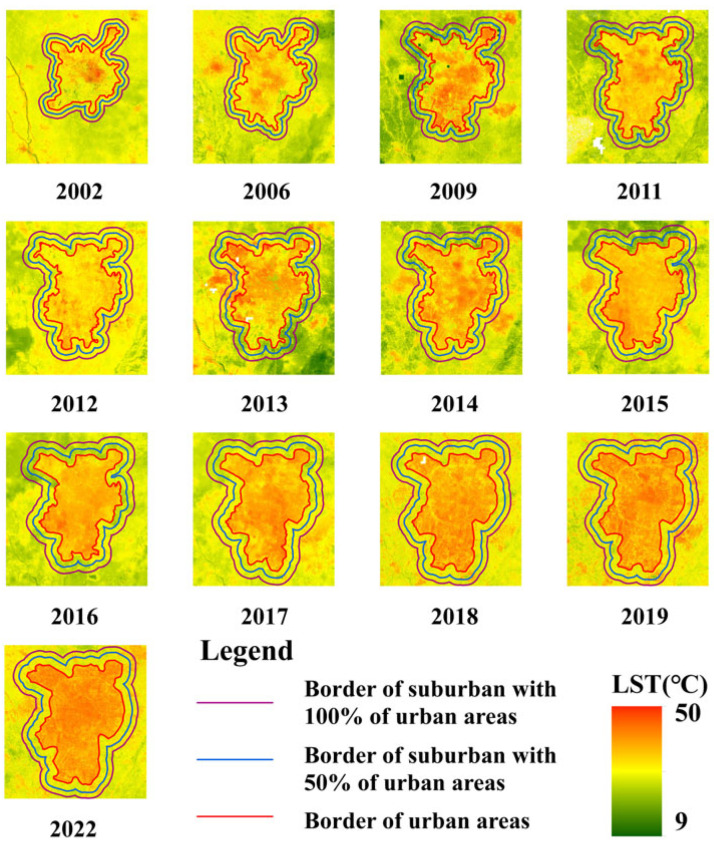
Spatial distribution of the summer averaged 30 m predicted LST using the proposed method from 2002 to 2022.

Both averaged summer LSTs from 2002 to 2022 were computed (Figure 11). As shown in Figure 11, due to the rapid urbanization, the spatial distribution of the LSTs showed an irregular distribution, and the high LST areas changed from urban to suburban areas. The high LST areas were mainly concentrated in the urban high-density blocks, where buildings and population were highly concentrated. The low LST areas were mainly concentrated in the mountainous areas of the suburban, such as the Longquan Mountain Range in the southeast of the study area. The spatial resolution characteristics of the LSTs from 2002 to 2022 were similar, such as the high LST areas covered by the built-up areas. The low LST areas were mainly distributed in the vegetation-covered areas, such as farmland and mountain areas outside the built-up areas. We can see that the increase in the SUHI effect was roughly in the “southeast-northwest” direction as the urban built-up area expanded. The expansion of the SUHI effect corresponds to the urban spatial growth pattern.

**Figure 12:**
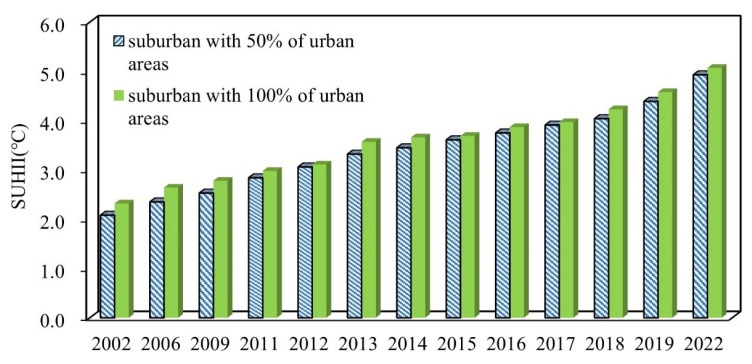
Temporal changes of SUHII in the study area from 2002 to 2022.

Figure 12 shows the summer SUHII from 2002 to 2022. The significantly increasing trends of the summer SUHII in Chengdu used the averaged 30 m predicted LSTs. The highest SUHII for summer occurred in 2022 (5.07 °C from a larger suburban area and 4.93 °C from a smaller suburban area). Summer SUHII increased from 2.32 °C in 2002 to 5.07 °C for a larger suburban area and increased by 2.85 °C in the same period for a smaller suburban area. This indicates a large SUHII in the summer from 2002 to 2022 over the Chengdu City. It not only increases the risk of heatwave extreme events but also presents a big challenge for scientists to mitigate serious SUHI effects.

### 3.4. Relationship between SUHI and Potential Driving Factors

**Figure 13:**
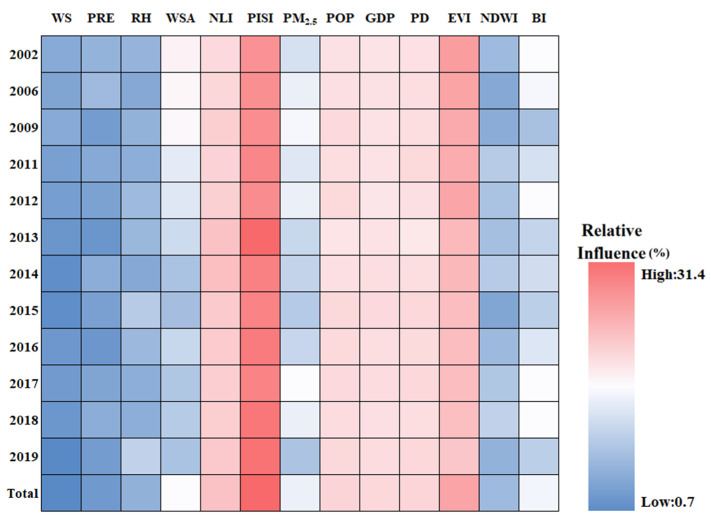
The relative influence of SUHI of the driving factors in Chengdu City from 2002 to 2019.

**Figure 14:**
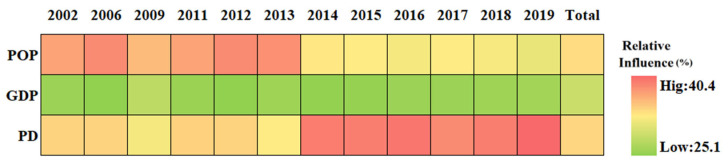
The relative influence of SUHI of the population shift driving factors in Chengdu City from 2002 to 2019.

**Figure 15:**
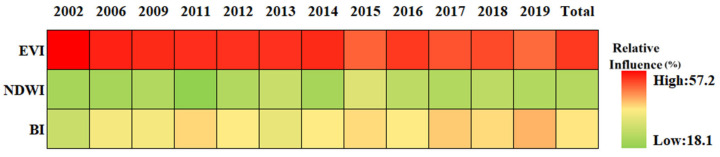
The relative influence of SUHI of the natural land surfaces driving factors in Chengdu City from 2002 to 2019.

**Figure 16:**
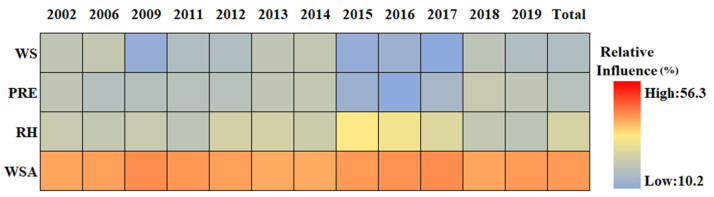
The relative influence of SUHI of the climate driving factors in Chengdu City from 2002 to 2019.

**Figure 17:**
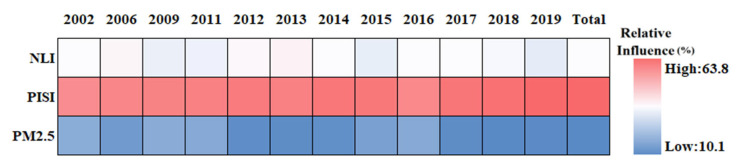
The relative influence of SUHI of the anthropogenic activity driving factors in Chengdu City from 2002 to 2019.

As described in Table 2, thirteen driving factors were selected to evaluate their influence on summer SUHI. The driving factors were divided into climate driving factors, anthropogenic activity driving factors, population shift driving factors, and natural land surfaces driving factors. Figure 13 presents the results of a BRT analysis for Chengdu City. The relative influence of each factor is scaled as a percentage [77]. Overall, on average the most important factors are PISI, EVI, and NLI, with about 26.9%, 17.4%, and 12.5%, respectively. The other influences range from high to low are POP, PD, GDP, WSA, BI, PM_2.5_, NDWI, RH, PRE, and WS, with 9.7%, 9.5%, 9.0%, 3.1%, 3.0%, 2.9%, 1.9%, 1.8%, 1.4%, and 1.1% on average, respectively. For the population shift driving factors (Figure 14), the natural land surfaces driving factors (Figure 15), the climate driving factors (Figure 16), and the anthropogenic activity driving factors (Figure 17), PD, EVI, WSA, and PISI are the most influential factors with the influence of 37.6%, 50.1%, 50.8, and 59.2%, respectively.

**Figure 18:**
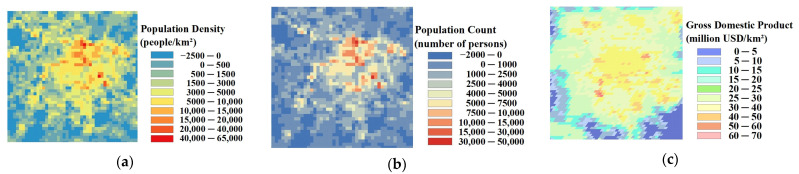
Spatial and temporal changes in PD, POP, and GDP in Chengdu City from 2002 to 2019. (**a**) population density (PD); (**b**) population count (POP); (**c**) gross domestic product (GDP).

Overall, each one of the potential driving factors had a comparable influence on SUHI. EVI has been widely used to characterize vegetation coverage. Previous studies have shown that SUHII is negatively correlated with EVI across 419 global big cities [78]. From 2002 to 2019, the relative influence of SUHI on EVI is gradually weakening, owing to human activities. The contribution of PISI and NLI was relatively high, indicating that the built-up area and economic development are the main causes of SUHI, while the influence of the climate factors is relatively low during the study period. The results show that the relative influence of SUHI on the climate factors may not be significant. Therefore, the intensification of human activities and economic activities is the main reason for the aggravation of the SUHI effect in Chengdu. As shown in Figure 18, from 2002 to 2019, PD, POP, and GDP increased in Chengdu City and were mainly concentrated in the urban areas. This was mainly due to the increasing centralization of the city, with various industrial zones expanding around the city center.

## 4. Discussion

An MGWR-CFSDAF spatiotemporal fusion method was proposed to generate high-spatiotemporal-resolution LST data from Landsat, HJ-1B, and MODIS. Although the proposed method can preserve spatial detail and generate high-resolution LST images with high accuracy in Chengdu City, there are also some limitations. Firstly, the performance of the proposed method greatly relies on the pairs of temporally close LST images, which only allows for the clear-sky conditions because the TIR data is difficult to obtain due to cloud cover [79,80,81]. If we want to acquire all-weather LST data, more effective cloud removal methods should be adopted to mitigate the influence of clouds. Secondly, since the overpass time of the Landsat, HJ-1B, and MODIS are different, within half an hour, a time normalization method should be applied to correct for possible inconsistencies in the future [82]. Thirdly, the spatial distribution of the LST is significantly influenced not only by variations in surface thermal properties but also by a pronounced terrain effect [83]. In this study, the spatiotemporal fusion accuracy of the LST data is less affected by mountainous terrain since the study area primarily comprises flat plains. However, this also suggests that further research is needed to explore whether the research method is applicable to urban areas with significant topographic variations. In addition, the main cause of urban thermal environmental change is carbon dioxide (CO_2_) [84]. The spatiotemporal distribution of CO_2_ emissions has been affected by land use/cover change (LUCC) [85]. Deng et al. [86] found that the potential changes in the LST were caused by LUCC. Therefore, in order to mitigate the SUHI effect and meet China’s target of carbon neutrality, future studies are needed to explore the relationships between urban expansion, land use changes, CO_2_ emissions, and the SUHI effect.

## 5. Conclusions

This paper took Chengdu, a typical cloudy and rainy city that easily satisfies the missing-filled satellite data scenario, as a case study for SUHII monitoring and performed quantitative analyses to investigate the influence of thirteen potential driving factors on the SUHII from 2002 to 2019. Firstly, high-spatiotemporal-resolution LST dataset with 30 m spatial resolution and 8-day temporal resolution were predicted by the proposed method using an MGWR coupling CFSDAF method. The performance of the method could generate high-accuracy summer LST datasets better than the CFSDAF, and FSDAF methods for the MOD11A2 dataset. Secondly, significantly increasing trends in the SUHII in Chengdu from 2.32 °C in 2002 to 5.07 °C in 2022 were observed for larger suburban areas and increased 2.85 °C during the same period for a smaller suburban area. Finally, PISI, EVI, and NLI are the three most influential factors on SUHI. The total contribution for the driving factors (PISI > EVI > NLI > POP > PD > GDP > WSA > BI > PM_2.5_ > NDWI > RH > PRE > WS) indicated that the summer SUHI in Chengdu is highly affected by the anthropogenic factor. So, we recommend that the anthropogenic activity driving factor should be considered with CO_2_ emissions and land use changes for urban planning to mitigate the SUHI effect.

## Figures and Tables

**Table 1:** The characteristics of inputs Landsat, HJ-1B, MOD11A1 and MOD11A2 LST satellite LST data of the proposed method.

Year	LST Image Pair on *t*_1_ (a Prior Date)		MOD11A2 on *t*_2_ (the Prediction Date)
	Landsat—MOD11A1 Pair	HJ-1B—MOD11A1 Pair	
2002	25 June	/	7–14 April, 9–16 May, 25 May–1 June, 12–19 July, 21–28 August, 29 August–5 September, 6–13 September
2006	19 May	/	1–8 May, 17–24 May, 25 May–1 June, 18–25 June, 20–27 July, 28 July–4 August
2009	/	20 April	17–24 May, 2–9 June, 22–29 September
2011	/	23 August	17–24 May, 12–19 July, 5–12 August, 21–28 August, 29 August–5 September
2012	/	27 April	7–14 April, 22–29 April, 12–19 August
2013	/	20 April	7–14 April, 15–22 April, 17–24 May, 25 May–1 June, 2–9 June, 10–17 June, 13–20 August, 21–28 August
2014	/	28 July	7–14 April, 12–19 July, 20–27 July
2015	/	16 April	15–22 April, 1–8 May, 25 May–1 June, 12–19 July, 20–27 July, 5–12 August
2016	/	16 May	30 April–7 May, 8–15 May, 1–8 June, 11–18 July, 5–12 September
2017	1 May	/	28 July–4 August
2018	2 April	/	23–30 April, 20–27 July, 21–28 August, 29 August–5 September
2019	11 August	/	7–14 April, 23–30 April, 9–16 May, 2–9 June, 5–12 August, 21–28 August, 22–29 September
2022	21 April	/	7–14 April, 23–30 April, 4–11 July, 28 July–4 August, 5–12 August, 13–20 August

**Table 2:** The potential driving factors selected in this study.

Type	Name	Data Source	Description	Spatial/Temporal Resolution
Climate	The satellite precipitation product (PRE) [35,36]	http://www.cpc.ncep.noaa.gov/, accessed on 3 February 2023	PRE comes from the satellite precipitation data set (CMORPH) [37,38], it can produce global precipitation estimates.	0.25°/30 min
	Wind speed (WS) [39,40] Relative humidity (RH) [41]	https://cds.climate.copernicus.eu/, accessed on 8 February 2023	ERA5 is a climate reanalysis dataset developed by the European Centre for Medium-Range Weather Forecasts (ECMWF). It provides comprehensive and high-resolution information about various atmospheric parameters, including wind speed [42] and humidity [43] on a global scale.	0.25°/hourly
	White sky albedo (WSA) [44,45]	https://search.earthdata.nasa.gov/, accessed on 3 February 2023	WSA is measured or estimated from the MCD43A3 dataset [46,47] and is a parameter that describes the amount of solar radiation reflected by the Earth’s surface under overcast or white-sky conditions.	500 m/16 days
Anthropogenic activity	Nighttime light index (NLI) [48,49]	https://ngdc.noaa.gov/, accessed on 1 March 2023 https://ladsweb.modaps.eosdis.nasa.gov/, accessed on 25 February 2023	NLI utilizes nighttime light data from satellite observations to assess human activities and urbanization during the night. The DMSP-OLS (Defense Meteorological Satellite Program—Operational Linescan System) and NPP-VIIRS (National Polar-orbiting Partnership—Visible Infrared Imaging Radiometer Suite) [50,51] datasets supply the necessary nighttime light data for NLI calculation.	1000 m/monthly
	Perpendicular impervious surface index (PISI)	https://search.earthdata.nasa.gov/, accessed on 18 February 2023	PISI is a spectral index used to estimate impervious surfaces, such as roads, buildings, and pavements, from the blue band ($\rho_{blue}$) and near-infrared band ($\rho_{nir}$) of the MOD09A1 data. The formula for PISI can be expressed as follows [52,53]: $\mathrm{PISI}=0.8192\rho_{blue}\;-\;0.5735\rho_{nir}+0.075$	500 m/8 days
	PM_2.5_ [54,55]	https://zenodo.org/records/6398971, accessed on 19 February 2023	ChinaHighPM_2.5_ is a high-quality dataset in the CHAP series, providing comprehensive, high-res, long-term ground-level air pollutant data for China. Generated using AI and various data sources, it captures spatiotemporal air pollution variations, offering valuable insights into China’s air quality.	1000 m/monthly
Population shift	Population counts (POP)	https://hub.worldpop.org/, accessed on 11 March 2023	The population data we collected are two products of the WorldPop dataset [56]. They describe the residential population where they actually live.	1000 m/yearly
	Population density (PD) [57,58]			
	Gross domestic products (GDP) [59,60]	http://www.gis5g.com/, accessed on 16 March 2023	GDP is collected from the Geographic Data Sharing Infrastructure, global resources data cloud, which indicates the economic status within the city. Herein, the GDP density was selected to quantify surface urban heat island.	1000 m/yearly
Natural land surfaces change	Enhanced vegetation index (EVI) [61,62]	https://search.earthdata.nasa.gov/, accessed on 13 March 2023	EVI is derived from the MOD13A3 dataset [63,64,65], it can be used for a long-term spatiotemporal analysis of vegetation greenness over the global.	1000 m/monthly
	Bare-soil index (BI)	https://search.earthdata.nasa.gov/, accessed on 21 March 2023	BI is valuable for detecting and quantifying the amount of exposed bare soil, aiding in the assessment of land cover changes and other soil-related phenomena. It is estimated from the shortwave infrared ($\rho_{swir}$), red ($\rho_{red}$), near-infrared ($\rho_{nir}$), and blue ($\rho_{blue}$) bands of the MOD09A1 data. The formula for BI can be expressed as follows [66,67]: $\mathrm{BI}=\frac{\left(\left(\rho_{swir\;}+\rho_{red}\right)\;-\;\left(\rho_{nir}+\rho_{blue}\right)\right)}{\left(\left(\rho_{swir}+\rho_{red}\right)\;+\;\left(\rho_{nir}\;+\;\rho_{blue}\right)\right)}$	500 m/8 days
	Normalized difference water index (NDWI)	https://search.earthdata.nasa.gov/, accessed on 22 March 2023	NDWI is a remote sensing spectral index used to identify and assess the presence of water bodies in satellite imagery and other remotely sensed data. It quantifies the relative difference in reflectance between the near-infrared ($\rho_{nir}$) and green visible light ($\rho_{green}$) bands of the MOD09A1 data. The formula for NDWI can be expressed as follows [68,69]: $\mathrm{NDWI}=\frac{\rho_{green}\;-\;\rho_{nir}}{\rho_{green}\;+\;\rho_{nir}}$	500 m/8 days

**Table 3:** Classification accuracy of SVM, ANN and MLC from 2002 to 2022.

Year/Accuracy (%)	SVM		ANN		MLC	
	Overall Accuracy	Kappa Coefficient	Overall Accuracy	Kappa Coefficient	Overall Accuracy	Kappa Coefficient
2002	99.47	98.96	98.50	98.27	98.06	98.24
2006	98.22	98.01	98.18	97.68	97.48	97.52
2009	99.10	99.07	98.94	98.71	97.53	96.99
2011	99.23	99.47	99.01	98.20	96.44	96.00
2012	98.76	98.24	98.01	97.65	97.48	97.71
2013	98.59	99.05	97.48	97.25	97.48	97.09
2014	99.46	99.78	97.18	98.48	98.25	97.48
2015	99.05	99.26	98.36	98.04	98.10	98.02
2016	97.89	98.48	96.25	97.63	95.66	96.75
2017	98.19	97.79	97.50	98.64	96.57	96.28
2018	99.12	98.75	97.64	97.20	96.41	96.49
2019	97.45	97.21	96.48	97.96	95.75	95.06
2022	99.43	98.24	98.09	97.95	97.27	96.99

**Table 4:** Downscaling statistics for MGWR, GWR, and TsHARP method in this study.

Date	MGWR		GWR		TsHARP	
	RMSE	ME	RMSE	ME	RMSE	ME
25 June 2002	2.12	0.06	2.66	0.25	3.97	2.98
19 May 2006	2.18	0.32	3.25	0.69	−4.01	−2.35
20 April 2009	1.79	0.24	−2.66	1.58	6.25	2.64
23 August 2011	−2.59	−0.05	3.67	0.98	4.23	3.25
27 April 2012	4.65	0.07	5.32	−0.45	5.26	−0.26
20April 2013	1.26	0.25	1.98	−2.77	3.30	−3.06
28 July 2014	1.77	0.63	−2.06	1.35	−2.16	1.23
16 April 2015	2.06	0.54	3.17	0.66	2.59	1.54
16 May 2016	−1.59	−0.26	2.31	0.58	3.07	0.44
1 May 2017	3.65	−0.06	−4.65	0.09	3.76	3.99
2 April 2018	2.97	0.62	6.01	−0.89	−3.02	1.26
11 August 2019	−3.57	0.16	5.32	1.67	−5.60	−1.38
21 April 2022	2.64	0.48	−2.99	3.25	2.76	2.64

**Table 5:** Comparison of the predicted LSTs using the proposed method, classical FSDAF and MOD11A2, respectively, with in situ LSTs during the summer from 2002 to 2022.

Average Summer In Situ LST Acquisition Year	*R* ^2^			
	The Proposed Method	CFSDAF	FSDAF	MOD11A2
2002	0.9048 **	0.8991 **	0.8422 *	0.8130 *
2006	0.8925 **	0.8806 **	0.8616 **	0.7948
2009	0.9023 **	0.8595 *	0.8453 *	0.8246
2011	0.8779	0.8651	0.8660 *	0.8157
2012	0.8849 *	0.8022 *	0.7963	0.7850
2013	0.9091 **	0.8947 **	0.8526	0.8501 *
2014	0.8730 *	0.8546 *	0.8026	0.7584
2015	0.8815 *	0.8802 *	0.8730 *	0.8039
2016	0.9025 **	0.8928 *	0.8840 *	0.7964
2017	0.8661	0.8545	0.8532	0.8061
2018	0.8990 **	0.8920 *	0.8859 *	0.8712 *
2019	0.9065 **	0.8933 **	0.8953 **	0.7990
2022	0.9008 **	0.8654 *	0.7821	0.7605

Note: * = significant at *p* = 0.05, ** = significant at *p* = 0.001.

## Data Availability

The data presented in this study are available on request from the corresponding author. The data are not publicly available due to privacy protection reasons.

## References

[1] Naikoo M.W., Islam A.R.M.T., Mallick J., Rahman A. (2022). Land use/land cover change and its impact on surface urban heat island and urban thermal comfort in a metropolitan city. Urban Clim..

[2] Moazzam M.F.U., Doh Y.H., Lee B.G. (2022). Impact of urbanization on land surface temperature and surface urban heat Island using optical remote sensing data: A case study of Jeju Island, Republic of Korea. Build. Environ..

[3] Zhou D., Xiao J., Bonafoni S., Berger C., Deilami K., Zhou Y., Frolking S., Yao R., Qiao Z., Sobrino J.A. (2018). Satellite remote sensing of surface urban heat islands: Progress, challenges, and perspectives. Remote Sens..

[4] Zhou Y., Zhao H., Mao S., Zhang G., Jin Y., Luo Y., Huo W., Pan Z., An P., Lun F. (2022). Exploring surface urban heat island (SUHI) intensity and its implications based on urban 3D neighborhood metrics: An investigation of 57 Chinese cities. Sci. Total Environ..

[5] Lu R., Xu K., Chen R., Chen W., Li F., Lv C. (2023). Heat waves in summer 2022 and increasing concern regarding heat waves in general. Atmos. Ocean Sci. Lett..

[6] Zhang B., Chen H., Lu B. (2023). An Early Warning System for Heatwave-Induced Health Risks in China: A Sub-Seasonal to Seasonal Perspective—China, 2022. China CDC Wkly..

[7] Hao Z., Chen Y., Feng S., Liao Z., An N., Li P. (2023). The 2022 Sichuan-Chongqing spatio-temporally compound extremes: A bitter taste of novel hazards. Sci. Bull..

[8] Li H., Zhou Y., Li X., Meng L., Wang X., Wu S., Sodoudi S. (2018). A new method to quantify surface urban heat island intensity. Sci. Total Environ..

[9] Huang B., Wang J., Song H., Fu D., Wong K. (2013). Generating high spatiotemporal resolution land surface temperature for urban heat island monitoring. IEEE Geosci. Remote Sens. Lett..

[10] Wu P., Yin Z., Zeng C., Duan S.-B., Göttsche F.-M., Ma X., Li X., Yang H., Shen H. (2021). Spatially continuous and high-resolution land surface temperature product generation: A review of reconstruction and spatiotemporal fusion techniques. IEEE Trans. Geosci. Remote Sen..

[11] Yin Z., Wu P., Foody G.M., Wu Y., Liu Z., Du Y., Ling F. (2020). Spatiotemporal fusion of land surface temperature based on a convolutional neural network. IEEE Trans. Geosci. Remote Sens..

[12] Zhu X., Song X., Leng P., Li X., Gao L., Guo D., Cai S. (2021). A framework for generating high spatiotemporal resolution land surface temperature in heterogeneous areas. Remote Sens..

[13] Gao F., Masek J., Schwaller M., Hall F. (2006). On the blending of the Landsat and MODIS surface reflectance: Predicting daily Landsat surface reflectance. IEEE Trans. Geosci. Remote Sens..

[14] Zhukov B., Oertel D., Lanzl F., Reinhackel G. (1999). Unmixing-based multisensor multiresolution image fusion. IEEE Trans. Geosci. Remote Sens..

[15] Choe Y.-J., Yom J.-H. (2020). Improving accuracy of land surface temperature prediction model based on deep-learning. Spat. Inf. Res..

[16] Li S., Wang J., Li D., Ran Z., Yang B. (2021). Evaluation of Landsat 8-like land surface temperature by fusing Landsat 8 and MODIS land surface temperature product. Processes.

[17] Liu X., Zhou Y., Yue W., Li X., Liu Y., Lu D. (2020). Spatiotemporal patterns of summer urban heat island in Beijing, China using an improved land surface temperature. J. Clean. Prod..

[18] Shen H., Huang L., Zhang L., Wu P., Zeng C. (2016). Long-term and fine-scale satellite monitoring of the urban heat island effect by the fusion of multi-temporal and multi-sensor remote sensed data: A 26-year case study of the city of Wuhan in China. Remote Sens. Environ..

[19] Zhu X., Chen J., Gao F., Chen X., Masek J.G. (2010). An enhanced spatial and temporal adaptive reflectance fusion model for complex heterogeneous regions. Remote Sens. Environ..

[20] Weng Q., Fu P., Gao F. (2014). Generating daily land surface temperature at Landsat resolution by fusing Landsat and MODIS data. Remote Sens. Environ..

[21] Wu P., Shen H., Zhang L., Göttsche F.-M. (2015). Integrated fusion of multi-scale polar-orbiting and geostationary satellite observations for the mapping of high spatial and temporal resolution land surface temperature. Remote Sens. Environ..

[22] Zhao G., Zhang Y., Tan J., Li C., Ren Y. (2020). A data fusion modeling framework for retrieval of land surface temperature from Landsat-8 and MODIS Data. Sensors.

[23] Huang B., Song H. (2012). Spatiotemporal reflectance fusion via sparse representation. IEEE Trans. Geosci. Remote Sens..

[24] Zhu X., Helmer E.H., Gao F., Liu D., Chen J., Lefsky M.A. (2016). A flexible spatiotemporal method for fusing satellite images with different resolutions. Remote Sens. Environ..

[25] Zhao Y., Huang B., Song H. (2018). A robust adaptive spatial and temporal image fusion model for complex land surface changes. Remote Sens. Environ..

[26] Shi C., Wang N., Zhang Q., Liu Z., Zhu X. (2022). A Comprehensive Flexible Spatiotemporal Data Fusion Method (CFSDAF) for Generating High Spatiotemporal Resolution Land Surface Temperature in Urban Area. IEEE J. Sel. Top. Appl. Earth Obs. Remote Sens..

[27] Sobrino J., Oltra-Carrió R., Sòria G., Bianchi R., Paganini M. (2012). Impact of spatial resolution and satellite overpass time on evaluation of the surface urban heat island effects. Remote Sens. Environ..

[28] Luo P., Yu B., Li P., Liang P., Liang Y., Yang L. (2023). How 2D and 3D built environments impact urban surface temperature under extreme heat: A study in Chengdu, China. Build. Environ..

[29] Jiménez-Muñoz J.C., Sobrino J.A. (2003). A generalized single-channel method for retrieving land surface temperature from remote sensing data. J. Geophys. Res..

[30] Wu H., Ye L.-P., Shi W.-Z., Clarke K.C. (2014). Assessing the effects of land use spatial structure on urban heat islands using HJ-1B remote sensing imagery in Wuhan, China. Int. J. Appl. Earth Obs. Geoinf..

[31] Chen B., Wu Z., Wang J., Dong J., Guan L., Chen J., Yang K., Xie G. (2015). Spatio-temporal prediction of leaf area index of rubber plantation using HJ-1A/1B CCD images and recurrent neural network. ISPRS J. Photogramm..

[32] Chander G., Markham B.L., Helder D.L. (2009). Summary of current radiometric calibration coefficients for Landsat MSS, TM, ETM+, and EO-1 ALI sensors. Remote Sens Environ..

[33] Duan S.-B., Yan G.-J., Qian Y.-G., Li Z., Jiang X., Li X.W. (2008). Two single-channel algorithms for retrieving land surface temperature from the simulated HJ-1B data. Prog. Nat. Sci..

[34] Yoo C., Im J., Cho D., Yokoya N., Xia J., Bechtel B. (2020). Estimation of all-weather 1 km MODIS land surface temperature for humid summer days. Remote Sens..

[35] Wang J., Chen F., Doan Q.-V., Xu Y. (2021). Exploring the effect of urbanization on hourly extreme rainfall over Yangtze River Delta of China. Urban Clim..

[36] Zhong S., Qian Y., Zhao C., Leung R., Yang X.Q. (2015). A case study of urbanization impact on summer precipitation in the Greater Beijing Metropolitan Area: Urban heat island versus aerosol effects. J. Geophys. Res. Atmos..

[37] Gebregiorgis A.S., Hossain F. (2015). How well can we estimate error variance of satellite precipitation data around the world?. Atmos. Res..

[38] Chen S., Li W.-B., Du Y.-D., Mao C.-Y., Zhang L. (2015). Urbanization effect on precipitation over the Pearl River Delta based on CMORPH data. Adv. Clim. Chang. Res..

[39] Nogueira M., Hurduc A., Ermida S., Lima D.C., Soares P.M., Johannsen F., Dutra E. (2022). Assessment of the Paris urban heat island in ERA5 and offline SURFEX-TEB (v8. 1) simulations using the METEOSAT land surface temperature product. Geosci. Model Dev..

[40] Sultana S., Satyanarayana A. (2023). Impact of land use land cover on variation of urban heat island characteristics and surface energy fluxes using WRF and urban canopy model over metropolitan cities of India. Theoret. Appl. Climatol..

[41] Varentsov M., Konstantinov P., Repina I., Artamonov A., Pechkin A., Soromotin A., Esau I., Baklanov A. (2023). Observations of the urban boundary layer in a cold climate city. Urban Clim..

[42] Hersbach H., Bell B., Berrisford P., Hirahara S., Horányi A., Muñoz-Sabater J., Nicolas J., Peubey C., Radu R., Schepers D. (2020). The ERA5 global reanalysis. Q. J. R. Meteorol. Soc..

[43] Muñoz-Sabater J., Dutra E., Agustí-Panareda A., Albergel C., Arduini G., Balsamo G., Boussetta S., Choulga M., Harrigan S., Hersbach H. (2021). ERA5-Land: A state-of-the-art global reanalysis dataset for land applications. Earth Syst. Sci. Data Discuss..

[44] Karimi A., Mohammad P., Gachkar S., Gachkar D., García-Martínez A., Moreno-Rangel D., Brown R.D. (2021). Surface urban heat island assessment of a cold desert city: A case study over the Isfahan Metropolitan Area of Iran. Atmosphere.

[45] Du H., Zhan W., Voogt J., Bechtel B., Chakraborty T., Liu Z., Hu L., Wang Z., Li J., Fu P. (2023). Contrasting trends and drivers of global surface and canopy urban heat islands. Geophys. Res. Lett..

[46] Liu Z., Zhan W., Lai J., Bechtel B., Lee X., Hong F., Li L., Huang F., Li J. (2022). Taxonomy of seasonal and diurnal clear-sky climatology of surface urban heat island dynamics across global cities. ISPRS J. Photogramm. Remote Sens..

[47] Wu X., Wen J., Xiao Q., You D., Dou B., Lin X., Hueni A. (2018). Accuracy assessment on MODIS (V006), GLASS and MuSyQ land-surface albedo products: A case study in the Heihe River Basin, China. Remote Sens..

[48] Li J., Wang F., Fu Y., Guo B., Zhao Y., Yu H. (2020). A novel SUHI referenced estimation method for multicenters urban agglomeration using DMSP/OLS nighttime light data. IEEE J. Stars.

[49] Li C., Li X., Li T., Meng Q., Yu W. (2021). LMedS-based power regression: An optimal and automatic method of radiometric intercalibration for DMSP-OLS NTL imagery. IEEE J. Stars.

[50] Yang J., Wang Y., Xiu C., Xiao X., Xia J., Jin C. (2020). Optimizing local climate zones to mitigate urban heat island effect in human settlements. J. Clean. Prod..

[51] Sun Y., Wang S., Wang Y. (2020). Estimating local-scale urban heat island intensity using nighttime light satellite imageries. Sustain. Cities Soc..

[52] Zhang X., Liu L., Chen X., Xie S., Gao Y. (2019). Fine land-cover mapping in China using Landsat datacube and an operational SPECLib-based approach. Remote Sens..

[53] Tian Y., Chen H., Song Q., Zheng K. (2018). A novel index for impervious surface area mapping: Development and validation. Remote Sens..

[54] Chen Y., Ke X., Min M., Zhang Y., Dai Y., Tang L. (2022). Do We Need More Urban Green Space to Alleviate PM2. 5 Pollution? A Case Study in Wuhan, China. Land.

[55] Li Z., Guo H., Zhang L., Liang D., Zhu Q., Liu X., Zhou H. (2022). Time-Series Monitoring of Dust-Proof Nets Covering Urban Construction Waste by Multispectral Images in Zhengzhou, China. Remote Sens..

[56] Liu P., Wang Q., Zhang D., Lu Y. (2020). An improved correction method of nighttime light data based on EVI and WorldPop data. Remote Sens..

[57] Vergara D., Blanco A. (2023). Surface Urban Heat Islands and Related Health Risk in the Philippines: A Geospatial Assessment Using Modis Data. ISPRS J. Photogr. Remote Sens..

[58] Athukorala D., Murayama Y. (2021). Urban heat island formation in Greater Cairo: Spatio-temporal analysis of daytime and nighttime land surface temperatures along the urban–rural gradient. Remote Sens..

[59] Chen J., Gao M., Cheng S., Hou W., Song M., Liu X., Liu Y. (2022). Global 1 km × 1 km gridded revised real gross domestic product and electricity consumption during 1992–2019 based on calibrated nighttime light data. Sci. Data.

[60] Jia W., Zhao S. (2020). Trends and drivers of land surface temperature along the urban-rural gradients in the largest urban agglomeration of China. Sci Total Environ..

[61] Wu X., Wang G., Yao R., Wang L., Yu D., Gui X. (2019). Investigating surface urban heat islands in South America based on MODIS data from 2003–2016. Remote Sens..

[62] Wu X., Xu Y., Chen H. (2020). Study on the spatial pattern of an extreme heat event by remote sensing: A case study of the 2013 extreme heat event in the Yangtze River Delta, China. Sustainability.

[63] Yao R., Wang L., Huang X., Chen X., Liu Z. (2019). Increased spatial heterogeneity in vegetation greenness due to vegetation greening in mainland China. Ecol. Indic..

[64] Jaafar H.H., Ahmad F.A. (2015). Crop yield prediction from remotely sensed vegetation indices and primary productivity in arid and semi-arid lands. Int. J. Remote Sens..

[65] Matsushita B., Yang W., Chen J., Onda Y., Qiu G. (2007). Sensitivity of the enhanced vegetation index (EVI) and normalized difference vegetation index (NDVI) to topographic effects: A case study in high-density cypress forest. Sensors.

[66] Wang G., Wang J., Zou X., Chai G., Wu M., Wang Z. (2019). Estimating the fractional cover of photosynthetic vegetation, non-photosynthetic vegetation and bare soil from MODIS data: Assessing the applicability of the NDVI-DFI model in the typical Xilingol grasslands. Int. J. Appl. Earth Obs. Geoinf..

[67] Diek S., Fornallaz F., Schaepman M.E., De Jong R. (2017). Barest pixel composite for agricultural areas using landsat time series. Remote Sens..

[68] Bisquert M., Sánchez J.M., Caselles V. (2014). Modeling fire danger in Galicia and Asturias (Spain) from MODIS images. Remote Sens..

[69] Zheng Y., Tang L., Wang H. (2021). An improved approach for monitoring urban built-up areas by combining NPP-VIIRS nighttime light, NDVI, NDWI, and NDBI. J. Clean. Prod..

[70] Arshad A., Zhang W., Zhang Z., Wang S., Zhang B., Cheema M.J.M., Shalamzari M.J. (2021). Reconstructing high-resolution gridded precipitation data using an improved downscaling approach over the high altitude mountain regions of Upper Indus Basin (UIB). Sci Total Environ..

[71] Yang C., Zhan Q., Lv Y., Liu H. (2019). Downscaling land surface temperature using multiscale geographically weighted regression over heterogeneous landscapes in Wuhan, China. IEEE J. Sel. Top. Appl. Earth Obs. Remote Sens..

[72] Li Y., Baorong Z., Xiaohong X., Zijun L. (2022). Application of a semivariogram based on a deep neural network to Ordinary Kriging interpolation of elevation data. PLoS ONE.

[73] Gu H., Wang J., Ma L., Shang Z., Zhang Q. (2019). Insights into the BRT (Boosted Regression Trees) method in the study of the climate-growth relationship of Masson pine in subtropical China. Forests.

[74] Zhang W., Du Z., Zhang D., Yu S., Hao Y. (2016). Boosted regression tree model-based assessment of the impacts of meteorological drivers of hand, foot and mouth disease in Guangdong, China. Sci. Total Environ..

[75] Sayegh A., Tate J.E., Ropkins K. (2016). Understanding how roadside concentrations of NOx are influenced by the background levels, traffic density, and meteorological conditions using Boosted Regression Trees. Atmos. Environ..

[76] Hu B., Xu Y., Huang X., Cheng Q., Ding Q., Bai L., Li Y. (2021). Improving urban land cover classification with combined use of sentinel-2 and sentinel-1 imagery. ISPRS Int. J. Geo-Inf..

[77] Carslaw D.C., Taylor P. (2009). Analysis of air pollution data at a mixed source location using boosted regression trees. Atmos. Environ..

[78] Peng S., Piao S., Ciais P., Friedlingstein P., Ottle C., Bréon F.-M., Nan H., Zhou L., Myneni R.B. (2012). Surface urban heat island across 419 global big cities. Environ. Sci. Technol..

[79] Cao R., Chen Y., Chen J., Zhu X., Shen M. (2020). Thick cloud removal in Landsat images based on autoregression of Landsat time-series data. Remote Sens. Environ..

[80] Xu M., Deng F., Jia S., Jia X., Plaza A.J. (2022). Attention mechanism-based generative adversarial networks for cloud removal in Landsat images. Remote Sens. Environ..

[81] Long D., Yan L., Bai L., Zhang C., Li X., Lei H., Yang H., Tian F., Zeng C., Meng X. (2020). Generation of MODIS-like land surface temperatures under all-weather conditions based on a data fusion approach. Remote Sens. Environ..

[82] Zhao W., Wu H., Yin G., Duan S.-B. (2019). Normalization of the temporal effect on the MODIS land surface temperature product using random forest regression. ISPRS J. Photogramm..

[83] Zhao W., Duan S.-B., Li A., Yin G. (2019). A practical method for reducing terrain effect on land surface temperature using random forest regression. Remote Sens. Environ..

[84] Liu G., Zhang F. (2022). How do trade-offs between urban expansion and ecological construction influence CO_2_ emissions? New evidence from China. Ecol. Indic..

[85] Liu G., Zhang F. (2022). Land Zoning Management to Achieve Carbon Neutrality: A Case Study of the Beijing–Tianjin–Hebei Urban Agglomeration, China. Land.

[86] Deng X., Zhao C., Lin Y., Zhang T., Qu Y., Zhang F., Wang Z., Wu F. (2014). Downscaling the impacts of large-scale LUCC on surface temperature along with IPCC RCPs: A global perspective. Energies.

